# Making Sense of Quorum Sensing at the Intestinal Mucosal Interface

**DOI:** 10.3390/cells11111734

**Published:** 2022-05-24

**Authors:** Friederike Uhlig, Niall P. Hyland

**Affiliations:** Department of Physiology and APC Microbiome Ireland, University College Cork, T12 XF62 Cork, Ireland; fuhlig@ucc.ie

**Keywords:** AHL, enteric nervous system, intestinal epithelium, intestinal disease, microbiome

## Abstract

The gut microbiome can produce metabolic products that exert diverse activities, including effects on the host. Short chain fatty acids and amino acid derivatives have been the focus of many studies, but given the high microbial density in the gastrointestinal tract, other bacterial products such as those released as part of quorum sensing are likely to play an important role for health and disease. In this review, we provide of an overview on quorum sensing (QS) in the gastrointestinal tract and summarise what is known regarding the role of QS molecules such as auto-inducing peptides (AIP) and acyl-homoserine lactones (AHL) from commensal, probiotic, and pathogenic bacteria in intestinal health and disease. QS regulates the expression of numerous genes including biofilm formation, bacteriocin and toxin secretion, and metabolism. QS has also been shown to play an important role in the bacteria–host interaction. We conclude that the mechanisms of action of QS at the intestinal neuro–immune interface need to be further investigated.

## 1. Introduction

**Figure 1:**
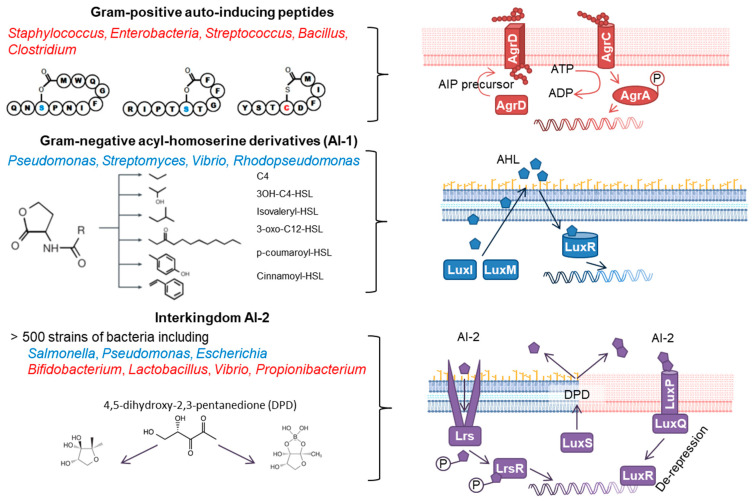
Schematic representation of the different quorum sensing molecules and intra-cellular signalling pathways Gram-positive (red) and Gram-negative (blue) bacteria. The QS molecule auto-inducer 2 (AI-2) has been described in both Gram-positive and Gram-negative bacteria (violet). Elements adapted by permission from [12], Springer Nature [13], and from BioRender.com (accessed on 2 November 2021).

Quorum sensing (QS) is defined as the ability to detect and respond to changes in population density. This process is particularly important for bacteria that undergo profound phenotypical changes when switching between different stages of growth (lag phase, exponential phase, and stationary phase), but equally occur in mammalian cells, especially in epithelial cells, cancer cells, immune cells, and stem cells [1,2,3,4]. Bacteria produce diffusible molecules to signal population density and the density-dependent increase of their concentration activates intracellular signalling pathways that lead to changes in gene expression (Figure 1, Table 1). The chemical nature of QS molecules is diverse and includes peptides (auto-inducing peptides, AIP; in Gram-positive bacteria), amphiphilic molecules (acyl-homoserine lactones, AHL, consisting of the amino acid derivative homoserine lactone (HSL) and fatty acids of different length in Gram-negative bacteria) and derivates of 4,5-dihydroxy-2,3-pentansione (DPD, in Gram-positive and -negative bacteria. Many species/strains produce relatively unique derivatives of AIP and AHL that allows the distinct signalling and crosstalk between bacteria [5,6,7].

### 1.1. Bacterial QS Signals

In Gram-positive bacteria, the QS machinery often consists of two-component systems (TCS). These are composed of the membrane-bound sensor histidine kinase that is activated by extracellular QS cues and catalyses the phosphorylation of an intracellular response regulator. The phosphorylated regulator affects target gene expression either directly through binding to their promotor or indirectly through the expression of regulatory RNA (RNAIII). This pathway is exemplified in Figure 1. Alternatively, Gram-positive bacteria, such as *Enterococcus*, *Streptococcus*, and *Clostridium*, also possess intracellular receptors for QS molecules (RRNPP family; Rap, Rgg, NprR, PlcR, and PrgX) [8,9,10,11,12]. These rely on peptide transport through the bacteria cell wall and regulate gene expression either directly or through the modulation of the activity of transcription factors.

In contrast, most Gram-negative bacteria utilize non-peptide N-Acyl homoserine lactones (AHL) for intra-species communication [13]. These are produced by AHL synthetases (LuxI or LuxM) from S-adenosylmethionine and cross the bacterial cell membrane due to their chemical nature and bind to intracellular receptors (LuxR) where they regulate the expression of target genes once activated by their respective ligand.

In addition to these exclusive systems, Gram-positive and Gram-negative bacteria both produce auto-inducer 2 (AI-2) through the AI synthesase LuxS. AI-2 and other AIs are thought to facilitate inter-species communication. It should be mentioned here that QS receptors of the LuxR family can also serve as inter-species sensors in some bacteria. In contrast to the classical LuxIR system, LuxR homologues exist in bacteria that do not produce species-specific AHL themselves. The expression of these homologues, LuxR solo, such as SdiA in *E. coli* and *S. typhimurium*, enables these bacteria to respond to QS signals from other bacteria. In addition to these three main groups, several bacterial species have been shown to produce rather unique QS molecules with distinct chemical structures. Since these are mostly described for non-enteric bacteria, the reader is referred to excellent reviews published elsewhere [13,14,15].

The expression of many bacterial genes is regulated by QS in order to ensure the survival of the population within changing environments [16,17,18]. To date, the modulation of gene expression by QS has mostly been studied in pathogenic bacteria, but functional QS systems are also present in gut residents (commensal) or probiotic bacteria [19]. QS-regulated factors may thus have context-dependent beneficial or detrimental effects for the host (Table 1, Section 3 and Section 4).

**Table 1:** QS activates target genes that benefit bacteria and have dichotomous effects on host physiology.

	Bacteria	Positive for Host	Negative for Host
Toxins, antibiotics	Kill superfluous bacteria with limited viability.	Antimicrobials can inhibit the growth of pathogenic bacteria (novel antibiotics) [20,21].	Toxins cause severe disease through damaging the intestinal epithelium, activating immune cells and neurons (pain) [22].
Proteases	Increase nutrient availability.	Modulate nutrient pool in the gut through protein degradation for metabolisation/fermentation by bacteria and absorption by the host.	Degrade host mucins and immunoglobulins decreasing host defences [23].
Biofilm formation	Allows motility of otherwise immotile bacteria, provides protection, allows GI colonisation.	Enables the growth and presence of beneficial bacteria (‘niche’) [24,25,26].	Protects from elimination/targeting by immune cells [27].
Metabolic adaptation	Switch to metabolic pathways using ready-to-use substrates, metabolic slowing [28,29].	Depletion of nutrients for the growth of pathogenic bacteria, production of inhibitory metabolites [30].	Depletion of nutrients for the host and adaptation to mucus degradation [31].

Bacteria defective in QS signalling cannot easily colonize the gastrointestinal (GI) tract. This has been found in pathogenic, commensal, and probiotic strains. *Streptococcus gallolyticus* subsp. *gallolyticus* for example can only colonize the murine intestine when it is able to produce the bacteriocin-like peptides (blpA and blpB). Blp-deficient mutants did not persist in the intestine. This may have important implications for the host given that the presence of *Streptococcus gallolyticus* is associated with the occurrence of colorectal cancer [32]. Blp from S. *gallolyticus* leads to a depletion of the commensal *Enterococcus faecalis* that facilitates S. *gallolyticus* persistence and furthers the disease-promoting environment. Equally, it was found that the luxS mutant of *Lactobacillus rhamnosus* GG and *Bifidobacterium breve* UCC2003 displayed significantly less persistence in the murine GI tract compared to the wild-type strains. This was associated with a higher sensitivity to gastric juice and impaired the ability of the bacteria to acquire iron in the iron-limited intestinal conditions, respectively, and suggests that luxS-induced signalling is crucial for intestinal survival [33,34] and bacterial adhesion to intestinal cells [35]. AI-2 production by *Escherichia coli* has also been shown to be a crucial determinant of microbial composition after streptomycin treatment. The deletion of the AI-2 receptor, and thus accumulation of AI-2 in the GI tract, increased colonisation with Firmicutes compared to animals that received strains in which luxS was deleted [36]. The depletion of Firmicutes and the increased abundance of Bacteroidetes is usually observed after streptomycin treatment. This finding, in line with others, demonstrate that the inhibition of QS does not per se lead to reduced persistence. Xu et al. (2006) reported that the deletion of the luxS gene, which produces the QS molecule AI-2, increases biofilm formation and virulence of *Staphylococcus epidermidis* [37].

### 1.2. Host Molecular and Cellular Targets of Bacterial QS Signals

A strong link between host health and disease and the presence and/or absence of bacteria in the gastrointestinal (GI) tract has been documented. However, the exact mechanisms underlying the beneficial effects of certain microbial metabolites remain to be established. Pathogen-associated molecular pattern (PAMP) recognition receptors such as toll-like receptors (TLR) have been the focus of many studies. More recently, it has become clear that receptor families that are not traditionally thought to be involved in bacterial sensing, may have evolved to respond to bacterial signals including QS-associated molecules. It is also tempting to speculate that among the many orphan receptors in the human genome, at least some will also respond to bacterial products.

G protein-coupled receptors (GPCRs) have been identified as potential targets in facilitating bacteria–host interactions by microbial-derived molecules. They can be activated by various bacterial compounds and metabolites [38,39,40]. GPCRs constitute a major part of the human genome and are one of the major classes of proteins that can be targeted pharmacologically. Of the known GPCR’s, taste receptors (T2Rs), Mas-related G protein-coupled receptors (MRG), and formyl peptide receptors (FPR) have all been shown to be involved in bacterial sensing [41]. For example, Tizzano et al. (2010) demonstrated that AHL released by *Pseudomonas aeruginosa* and *Escherichia coli* activate nasal chemosensory cells through the bitter taste receptor, Tas2r38 [42]. Moreover, Pudir et al. (2019) found that mast cell responses to Gram-positive QS peptides (AIP) are dependent upon MRGPRX2 expression [43]. Additionally, it is likely that AHL and AIP also activate metabolite and fatty acid receptors either in their naïve form or after processing in the GI tract given that they constitute acyl derivatives of homoserine lactone (Figure 1) and short peptides.

Other non-GPCR targets have also been described in sensing bacterial QS signals. Most recently, AHL have been added to the list of activators of the cytosolic aryl hydrocarbon receptors (AHRs), which also respond to plant products, xenobiotics, indole metabolites, and short chain fatty acids. AHR activity is differently regulated by distinct QS molecules [44,45,46], and this may constitute an important function of the AHR in the regulation of the host metabolism by pathogenic and commensal bacteria [47,48]. The scaffolding protein IQGAP1 (IQ motif-containing GTPase-activating protein 1) constitutes another intracellular target of AHL [49,50]. IQGAP1 regulates the activity of binding proteins and is involved in maintaining the cytoskeleton, which plays an important role for bacterial pathogenesis, given that bacteria interface with the intestinal epithelium when transiting through the GI tract. Further, the family of transient receptor potential (TRP) ligand and temperature-gated ion channels are likely involved in host sensing of QS. So far, it has been demonstrated that TRPA1 can be activated by the endotoxin lipopolysaccharide (LPS) independent of TLR4 [51] and that TRPV1-expressing cells are responsive to *Staphylococcus aureus* virulence factors [52].

In addition to traditional receptor-type sensors, the lipid composition of the cell membrane and the expression of glycolipids and transmembrane proteins have also been implicated in QS sensing. It has been found that small amphipathic peptides secreted by pathogenic *Staphylococcus aureus* (Phenol-soluble modulins, Psm) preferentially disrupt liquid disordered membrane domains [53], leading to cytotoxicity, whereas a host-expressed a disintegrin and metalloproteinase (ADAM) 10 has been found to be required for the binding and multimerization of *S*. *aureus* α-hemolysin [54,55,56]. The selectivity of toxins secreted by *Clostridium botulinum*, *tetani* and *Vibrio cholerae* for neurons is related to the presences of certain gangliosides (glycosphingolipids), synaptic vesicle glycoprotein 2, and synaptotagmin at the cell membrane, and are required for binding and internalization of botulinum, tetanus, and cholera toxin [57,58]. A similar specificity has been observed for other toxins secreted by *Corynebacterium diphtheriae*, *Bacillus anthracis*, *Clostridium perfringens*, *Escherichia coli*, and *Salmonella enterica*. On the contrary, few studies have investigated the effects of toxins and antimicrobial peptides secreted by commensal bacteria on the host. These so-called bacteriocins and bacteriocin-like peptides (BLP) are secreted by *Lactobacillus* and *Escherichia* species [21,59] and may represent novel antimicrobials. Although these substances do not seem to have equally strong toxic effects on host cells as those derived from pathogenic bacteria, it remains to be investigated whether they can affect host cellular signalling pathways. It has been shown that bacteriocins, can cross epithelial barriers in-vitro and potentially in-vivo [60,61], which might suggest that they have a physiological role to play beyond the GI tract.

The molecular targets described above are expressed by a multitude of cell types within the mucosal neuro–immune interface. Albeit intestinal epithelial cells would appear to be ideally located to sense bacterial metabolites in the GI tract, studies suggest that neurons and immune cells within the mucosa also directly respond to those molecules. This is possible through the crossing of such molecules across the epithelium [60,61,62] or bacterial cell translocation either into the epithelial cell or into the sub-epithelium [63,64,65,66]. Indeed, it has been suggested that molecules with a molecular weight below 7 KDa can cross the epithelial barrier [62]. This includes nearly all AHL and AIP as well as QS-associated molecules and has been shown for bacteriocins in an epithelial in-vitro system. Specific neurotoxins from *Clostridia* have evolved to translocate the intestinal wall through binding to proteins that are actively transported across the epithelium [57]. Pathogenic bacteria, such as *Salmonella typhimurium*, can survive and replicate intracellularly in epithelial and immune cells before inducing cell death, and, thus, can release secreted metabolites into deeper tissue layers. Further, *Staphylococcus aureus* can evade elimination by the immune system leading to re-occurring and chronic infection [63,64,65]. More recently, it has been shown that gut-resident bacteria are also capable of translocating the epithelial barrier and disseminating systemically [66], potentially by re-shaping the epithelial cytoskeleton through QS molecules [50].

## 2. Factors Influencing QS in the Gastrointestinal Tract

Traditionally, QS activation is associated with a high bacterial density but other signals including environmental stress, starvation, pH, and bile acids can induce changes in bacterial expression profiles.

### 2.1. Environmental Conditions (pH)

During their transfer through the human intestine, bacteria encounter different pH conditions. Gastric acid reduces the pH in the stomach to about 2–5, which constitutes a significant inhibitory barrier for most bacteria. Distal of the stomach, *Lactobacillus* and *Bifidobacterium* species can produce lactic acid and short chain fatty acids, respectively, which contribute to pH regulation of the intestine [67,68,69]. Acidic pH activates QS in *Lactobacillus rhamnosus* for example, and this is associated with increased bacteriocin production [70,71,72,73,74]. It has also been noted that acidic supernatants of *Lactobacillus* strains prevent biofilm formation by the pathogen *Pseudomonas aeruginosa* more so than neutralized supernatants. This suggests that it is the acidic pH itself that regulates the virulence of this pathogen. It cannot be excluded that yet uncharacterised mechanisms also play a role. [75]. This pH-dependent regulation is thought as an adaptation for pathogens to save energy in harsh conditions and to survive transit through the intestine [76]. In *Clostridium perfringens*, organic and inorganic acids induce the production of a self-quorum quenching molecule that inhibits QS [77]. Similar observations have been made for *Staphylococcus aureus* [78]. In contrast, the well-known stomach-residing pathogen *Helicobacter pylori* expresses an acid-detoxifying enzyme, Urease, to degrade stomach acid [79] while in *Streptococcus pyogenes*, acidification leads to conformational changes of the QS peptide receptor that allows binding of the peptide leading to the activation of virulence factor production [80].

### 2.2. Short Chain Fatty Acids

Short chain fatty acids (SCFAs) are produced by bacteria residing within the gastrointestinal tract through the metabolization of dietary starches and also proteins [81]. The predominant SCFAs in the GI tract are acetate, propionate, and butyrate, which make up 90–95% of the total short acid pool in the colon and faecal samples. The highest concentration (up to 100 mM) of SCFA can be found in the caecum [82] with an average contribution of 60:20:20 per cent for acetate, propionate, and butyrate, respectively [83]. Beyond constituting important energy sources for epithelial cells and contributing to gut immunity and gut–brain signalling for the host [84], SCFAs have also been found to modulate virulence factors and biofilm production of bacteria [85]. Whilst SCFA can reduce biofilm formation and QS activity [86,87] and, at high concentrations, inhibit bacterial growth [88], a number of pathogens appear to have adapted to the presence of SCFA in the GI tract and increase virulence factors production when exposed to acetate, propionate, or butyrate [85,89,90]. Interestingly, it has been found that SCFA also increase QS-regulated bacteriocin production the probiotic *Lactobacillus* species [73].

### 2.3. Dietary Compounds (Secondary Plant Products)

Numerous dietary molecules with diverse structures have been shown to interfere with bacterial virulence, including inhibition of QS [91,92,93,94]. They can compete with bacterial QS molecules for receptor binding as a result of structural similarities (pyrogallol) [95], sequester bacterial QS molecules (polyphenols and lignans) [96,97], inhibit QS molecule synthesis (naringenin) [98,99], accelerate QS molecule or receptor degradation (halogenated furanones) [100], and modulate receptor activity (cinnamaldehyde and luteolin) [101,102]. Additionally, plant products interfere with bacterial membranes and their metabolism leading to a decrease in QS molecule production. The precise mechanism of action for these plant-derived molecules remains to be completely understood. Further, most studies have focused on inhibiting pathogenic QS and QS-associated phenotypes such as biofilm formation. For example, Cho et al. found that out of extracts from 522 plants, only extracts from three Carex species (grass-like plants) exhibited a strong inhibitory effect on biofilm formation of *Pseudomonas aeruginosa* [103]. However, it is likely that plant products inhibiting QS in pathogenic bacteria may well simultaneously target commensal QS that may have subsequent effects on the host.

### 2.4. Host–Gut Microbial Co-Metabolism (Bile Acids)

The host produces primary bile acids in the liver and secretes bile salts (glycine or taurine of cholic acid and chenodeoxycholic acid) into the duodenum to enable the digestion of dietary fats. Thus, the concentration of bile acids is high in the proximal intestine and decreases along the ileum where the majority is resorbed (enterohepatic cycle) and metabolized by bacteria (to secondary bile acids such as deoxycholic acid and lithocholic acid). It has been shown that these different moieties have diverging effects on bacteria, for example by affecting *Clostridium difficile* survival. While host (predominantly primary) bile acids enhance bacterial growth and virulence, secondary bile acids have the opposite effect [104,105]. In many other pathogenic bacteria, exposure to bile acids triggers the production of QS signals and has been shown to increase biofilm formation, exopolysaccharide production, and antibiotic resistance [32,106]. In *Vibrio cholerae* for example, bile salts impair the DNA binding capacity of the QS receptor VqmA (repressor), which subsequently increases the production of virulence related genes [76]. Porcine bile extract similarly affects QS systems in non-pathogenic bacteria. In *Bifidobacteria*, exposure to bile acids increases bacterial growth and biofilm formation in a LuxS-dependent manner [107], and in some *Lactobacillus* species bile as well as individual bile acids increases AI-2 activity and adhesion to epithelial cells in *L. rhamnosus*, respectively [74]. In *L. plantarum*, higher LuxS activity is related to a higher resistance to bile acids and the production of anti-microbial substances [33]. For other *Lactobacillus* strains bile acids are toxic despite the high percentage of bile salt hydrolase producers in the *Lactobacillus* family [108].

### 2.5. Interkingdom Signalling Molecules

It has become clear that Gram-negative bacteria including *Escherichia coli*, *Shigella* sp., and *Salmonella* sp. express QseC, a predicted membrane-bound histidine sensor kinase, that enables them to respond to host-stress signals such as epinephrine and norepinephrine [109]. This inter-kingdom activation has been referred to as autoinducer-3 (AI-3) as it resembles the previously discussed AI systems and leads to the activation of QS gene expression. GI disease induced by a QseC-deficient mutant of an *E. coli*-like pathogen in rabbits, was significantly less severe than the wild type [110], and exposure of *Salmonella enterica* serovar typhi to epinephrine significantly increased hemolysin production [111]. Gram-positive bacteria including *Staphylococcus epidermidis* and *Enterococcus faecalis* also express QseC homologues, and activation by either epinephrine or norepinephrine induces biofilm formation [112].

Data also support a role for host serotonin in modulating QS activity and bacterial virulence [113,114]. Serotonin was found to increase virulence factor production by the Gram-positive *Pseudomonas aeruginosa*, leading to increased intestinal damage scores, and Lactate Dehydrogenase (LDH) and Myeloperoxidase (MPO) activity, all of which are markers of intestinal inflammation. The co-administration of serotonin with a QS-deficient *Pseudomonas aeruginosa* mutant was able to restore the impaired virulence of this deficient strain [114]. On the contrary, Kumar et al. reported that serotonin decreases the virulence of *Escherichia coli* EHEC and *Citrobacter rodentium* in-vitro and in mouse oral infection models. Strategies to increase serotonin concentration (serotonin transporter knock-out mice and selective serotonin reuptake inhibitor) prevented GI disease, whereas inhibition of the serotonin-producing enzyme tryptophan hydroxylase increased disease severity in this infection model [113]. This was associated with respective changes of virulence factor (espA, tir) expression.

### 2.6. Immunoglobulins

Secreted immunoglobulins (Ig) constitute important components of the host defence system. In the gut, it is specifically the dimeric IgA that is produced in both a T cell-dependent but also a T-cell-independent manner [115], and then secreted into the gut lumen. While a coating of bacteria and other “foreign” structures with other types of immunoglobulins labels them for recognition by phagocytes, IgA coating exerts more specific functions. It is particularly well suited for the agglutination of structures carrying target epitopes because of its dimeric structure and appears to control the metabolism of coated bacteria and prevent epithelial transition of potentially harmful agents rather than aiming at eliminating those through triggering an immune response [116]. Support for this notion comes from the observation that many commensal bacteria in the healthy gut are coated with IgA [117]. *Bacteroides thetaiotaomicron* induces the production of a specific IgA (mAb 225.4), that, when present in the lumen, decreases the expression of the respective antigen expressed by the bacterium. This is associated with a reduced pro-inflammatory transcriptional profile [118]. Furthermore, it has been suggested that an IgA coating might facilitate gut colonization through the crosslinking of bacteria with mucus or to each other to induce biofilm formation (which in many bacteria is regulated by QS) [116]. To date, however, it is unknown whether naturally induced IgA targets QS systems directly. On the other hand, it is hypothesized that the capacity of Ig to bind foreign structures could serve as a mechanism to quench QS signals as well as virulence factors. In this regard, attempts at developing specific antibodies to target QS molecules and toxins from *Staphylococcus aureus* and *Pseudomonas aeruginosa* have been made and are being developed as novel anti-virulence strategies [119,120,121].

## 3. QS at the Mucosal Neuroimmune Interface

**Figure 2:**
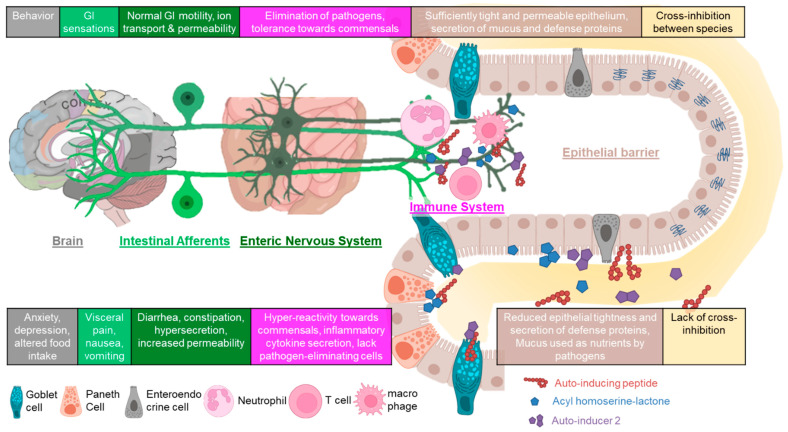
QS at the mucosal interface in health and disease. QS and associated molecules from commensal and probiotic bacteria (top table) play an important role in the development and maintenance of a healthy mucosa through modulating mucus secretion, epithelial barrier function (beige, Section 3.1), immune function (pink, Section 3.2), and neuronal activity (green, Section 3.3) in the gut. Pathogenic bacteria also produce QS molecules that can have harmful effects on mucosal neuro-immune function (bottom table). For simplification, the two plexi of the ENS have been depicted as one entity as have spinal and vagal intestinal afferents who provide input into the brain. For more details on the anatomy of the ENS and gut–brain signalling, the reader is referred to the references in Section 3.3. Figure elements from BioRender.com (accessed on 4 April 2022).

At the mucosal neuroimmune interface, it is epithelial cells, immune cells, and intestinal neurons that directly or indirectly interact with QS molecules. Given that those can originate from either pathogenic or commensal bacteria, the interaction with host cells may lead to either detrimental or beneficial effects (Figure 2).

### 3.1. Epithelial Barrier

Animals that develop without a microbiome (germfree) display differences in intestinal permeability and mucus structure that normalise upon colonisation [122,123]. Although it is currently unclear which mediators are responsible for these changes, it is likely that QS molecules play a role. Probiotics have been shown to enhance barrier function in-vitro and in animal studies [124]. However, with few exceptions, the molecules involved remain to be identified. In their study, Yan et al. (2007) identified the secreted proteins p40 and p75 in *Lactobacillus rhamnosus* GG supernatants that enhance epithelial survival in-vitro [125]. QS-regulated virulence factors from pathogenic *Citrobacterium difficile*, *E*. *coli*, and *S*. *typhimurium* decrease transepithelial resistance through the modulation of tight junctions via small Rho GTPase inhibition or activation [126,127]. It has further been shown that QS-associated molecules can negatively impact gut integrity through the activation of inflammatory pathways (NF-κB) that impair intestinal barrier function [128,129,130]. This predominantly negative association reported to date between QS-associated molecules and barrier function may reflect a bias toward studying pathogen-like bacteria for which QS is better understood rather than commensal organisms.

Pathogenic, commensal, and probiotic bacteria have all been shown to modulate the expression of mucins and other defence proteins in a manner that favours host resilience [131,132]. Mucins are produced by goblet cells located in the crypts of the epithelium. They are composed of a protein moiety that is heavily glycosylated, and, thus, forms a gel-like protective layer covering the epithelium [133]. *Lactobacillus* species potently induce mucin secretion in a colonic cell line via the production of a yet unidentified heat-resistant non-proteinaceous soluble mediator [134] and *Bifidobacterium longum* has been shown to increase the thickness of the mucin layer in vivo [135]. Equally, many pathogenic bacteria increase mucin secretion to shape their environmental ‘niche’ as it provides a sugar-rich source of nutrients and an intestinal adhesion scaffold [23,136,137]. Whilst it is clear that pathogenic QS-associated molecules are important for their effect on mucus, this remains to be investigated for commensal and probiotic bacteria.

The secretion of antimicrobial substances (human defence proteins, defensins, and lysozymes) from Paneth cells is another important mucosal defence mechanism that is modulated by bacteria. However, whether commensal bacteria and their QS systems play a role in Paneth cell section is still a matter of debate. On the one hand, regenerating islet-derived 3 γ (RegIIIγ) and IgA production are markedly reduced in germfree animals and α-defensin secretion can be induced in-vitro by exposing small intestinal crypts or cell lines to bacteria or bacterial products [138,139,140,141]. On the other hand, germfree mice also express defensin genes and are not devoid of Paneth cells, indicating that commensal bacteria are not required for defensin expression or differentiation of stem cells into Paneth cells [142]. Virulence factors produced by *Salmonella enterica* have been shown to reduce lysozyme granules and secretion in Paneth cells, which is counteracted by an increase in Paneth cell proliferation at later stages of the infection [143,144]. One possible explanation for these divergent findings could be that Paneth cell mediators are potentially deleterious for commensals and therefore, they regulate the turnover but not production of these mediators [145].

### 3.2. Immune Modulation

The GI tract harbours cells of both the innate and adaptive immune system, and both have been shown to be responsive to bacterial QS signals. AHL from pathogenic bacteria have been the prime focus of research in this area.

It has been found that AHL, especially 3-oxo-C12 HSL from *Pseudomonas aeruginosa*, can regulate the inflammatory response by acting as a chemoattractant for neutrophils [146] whilst then inhibiting their activity as well as that of macrophages and dendritic cells, subsequently inducing apoptosis in those cells. This serves to repress immune-mediated elimination of the pathogen [147,148]. A similar immune dampening effect has been described for infection with pathogenic *Yersinia pseudotuberculosis* and non-pathogenic *Salmonella typhimurium*. Infection with the pathogenic strain prior to the non-pathogenic strain led to the differentiation of macrophages into a tissue-protective phenotype [149].

Studies investigating the impact of the microbiota on the adaptive immune response have identified the transcription factor retinoic acid receptor (RAR)-related orphan-like (ROR)-γ in the sensing of commensal bacteria. ROR-γ is initially expressed by most lymphocytes in intestinal lymph follicles. During differentiation, only some subpopulations maintain a high expression of ROR-γ and those have been shown to be particularly responsive to signals from the intestinal microbiota. A major cell type that expresses high levels of ROR-γ are IL-17 secreting TH17 (CD4+) cells [150]. They are induced by specific nonculturable members of the microbiome, namely segmented filamentous bacteria (SFB) from the *cytophaga*-*flavobacter*-*bacteroides* group but not by *Enterococcus faecalis* or the altered Schaedler’s flora [150,151]. In animals colonised with SFB, the amount of AI-2 in mouse faeces is significantly higher than in germfree or SFB-naïve animals, and treatment with AI-2 increases the TH17 pool [152]. This AI-2-induced TH17 differentiation in-vivo appears to require serum amyloid A (SAA) proteins, a family of apoliproteins that are known to regulate several aspects of the immune system [152]. TH17 cells via IL-17 regulate recruitment and the phagocytic activity of neutrophils and play important roles for pathogen elimination and are associated with the ROR-γ^+^ innate lymphoid cells, ILC3. These have an inhibitory function on TH17 activity since their ablation induces low grade inflammation [153]. The second major ROR-γ^+^ T cell type constitutes a subpopulation of regulatory T cells (Treg). These cells acquire expression of the Treg marker FOXP3 and are particularly prominent in the small and large intestine [154]. It has been found that several strains of the class *Clostridia* as well as *Bacillus fragilis*, *Bacteroides thetaiotaomicron*, *Staphylococcus saprophyticus*, and *Clostridium rhamnosus* are the most prominent ROR-γ^+^ Treg cell-inducing bacteria. These strains are also capable of metabolising carbohydrates to SCFA [155,156,157]. FOXP3^+^ ROR-γ^+^ Treg cells produce either IL-10 or TGF-β, which inhibits TH17 and TH1 differentiation. Another Treg subpopulation (T1r) that does not express FOXP3, can be induced by *Bifidobacterium breve* and secretes the anti-inflammatory IL-10 [158]. Lastly, ROR-γ is expressed by populations of T cells that function in a similar manner to natural killer cells. Their abundance increases upon colonisation of germfree animals and subsequent IL-22 release contributes to the induction of antimicrobial peptides (AMP) in the intestinal epithelium [159]. To date, it has not been investigated whether ROR-γ directly responds to QS-regulated signals. It is interesting to note, however, that known ROR-γ ligands such as quinoline sulphonamide derivates and intermediates of the cholesterol biosynthetic pathway [160,161], which contain aromatic or sterol structures along with hydrophilic headgroups, are present in some AHL and other QS-regulated molecules.

Bacteria also induce a TH1 polarisation via T-bet activation whereas extracellular antigens and helminths induce the Gata3 transcription factor leading to a TH2 response. The main cytokines, IFN-γ and IL-5, activate macrophages and eosinophils, mast cells, and B cell differentiation, respectively [162].

### 3.3. Gut Intrinsic and Extrinsic Neural Function

Neurons are abundantly present in the GI tract. They comprise more than 100 million cells belonging to the intrinsic enteric nervous system (ENS) and receive dense innervation from extrinsic neurons [163]. Intrinsic enteric neurons are organised in a network underneath the mucosa (submucous plexus) and in between the muscle layers (myenteric plexus). The ENS constitutes chemically diverse neurons and glia cells, and regulate important GI functions such as fluid and ion homeostasis and motility [164]. Extrinsic neurons are located in ganglia outside the intestinal wall, either along the spinal cord (spinal afferents) or in the brain (vagal afferents), but extend their processes into all layers of the GI tract to relay information on the GI environment to the brain [165,166].

Studies using germfree mice and probiotic/antibiotic interventions have established an important function of the microbiota for GI function (ion transport and motility) as well as visceral pain and behaviour [167,168,169,170,171]. Germfree mice display an altered behavioural phenotype and stress response [172,173,174,175,176]. Some of these changes can be reversed by colonisation with a complete microbiota or non-pathogenic commensal strains, which suggests that these bacteria influence CNS function. Peripherally, *Lactobacillus* species were found to alter neuronal activity within the ENS [177,178] resulting in changes of GI motility, afferent nerve activity, and behaviour. However, the exact nature of this interaction and which metabolites regulate neuronal function remain in most cases unknown.

Early studies on bacteria–(enteric)neuron interactions have focussed on effects mediated by cell-wall components such as lipopolysaccharide and polysaccharide A [179,180,181]. However, it is becoming clear that secreted mediators from bacteria affect the ENS and sensory neurons as well. These include *Staphylococcus aureus* virulence factors [182,183], a polyketide molecule produced by *Mycobacterium ulcerans* [184], bacterial proteases [185], and a lipopeptide produced by *Escherichia coli* [186]. It is interesting to note that most of these mediators are regulated by QS signalling and reduce neuronal activity. In addition to these potential direct effects of QS mediators on neuronal function, it is likely that hormones and transmitters secreted by enteroendocrine cells in response to bacterial products also modulate intestinal neuron function [187].

## 4. QS and Association with Gastrointestinal Disease and Dysfunction

The importance of the bacterial QS system for the host’s well-being has overwhelmingly been studied in healthy animals and animal models of disease. These studies clearly show that AHL themselves as well as QS-regulated substances from (opportunistic) pathogens are important, if not essential, to cause disease. Indeed, the treatment of specific pathogen-free (SPF) mice with 3-o-C12-HSL significantly reduced body weight, induced GI inflammation, and increased GI permeability. This effect was recapitulated in germfree animals that received faecal microbiota transplants from 3-o-C12-HSL-treated animals, suggesting that 3-o-C12-HSL’s effects are mediated through the modulation of the microbiome [129]. In piglets, the concentration of specific AHL (3-o-C12-HSL, 3-o-C14-HSL) is associated with a low birth weight, intestinal damage, and a decreased abundance of most microbial communities that were investigated in this study [130]. Strategies blocking QS signalling (QS antagonists, quorum quenching, QS antibodies, and QS degradation) reduce disease severity in many animal models of infection [121,188]. In a *Pseudomonas aeruginosa* burn–infection model, treatment with the anti-virulence agent MvfR, an antagonist of QS receptor (PqsR), reduces intestinal inflammation and systemic inflammation, bacterial translocation, GI morphological changes, and the increased GI permeability that are associated with this model of disease [128]. Monoclonal antibodies against a designed hapten resembling a *Staphylococcus aureus* AIP, reduced lethality in an acute infection model [119] and many secondary plant products exert anti-virulence properties by inhibiting bacterial QS systems [91,93]. Carvacrol for example has been shown to reduce AI-2 activity of *Campylobacter jejuni* and *C. jejuni’s* persistence in the chicken gut [189]. It is suggested that Carvacrol binds and inhibits AHL synthase and thus, reduces the amount of available AHL [190].

Albeit less well understood, it is becoming evident that QS molecules are also important mediators in a human context. Landmann et al. (2018) found that up to 14 AHL can be detected in human stool and that one specific AHL (3-oxo-C12:2) was depleted in individuals with Inflammatory Bowel Disease (IBD) [191]. This particular AHL, in contrast to the related 3-oxo-C12, which is secreted by *Pseudomonas aeruginosa*, preserves the integrity of intestinal tight junctions and barrier function under inflammatory conditions in-vitro [147]. AI-2 has also been detected in stool as well as saliva from patients with IBD and healthy controls [192], and *Enterococcus* isolates from patients with IBD expressed more virulence and QS genes compared with the controls [193], suggesting that the quantification of QS molecules might constitute a novel biomarker of disease. Piewngam et al. (2018) found that intestinal colonisation with the pathogen *Staphylococcus aureus* was restricted to 25 participants of the *Bacillus* ssp negative subpopulation (99/200) of their study, suggesting that colonisation with *Bacillus* ssp regulates persistence and virulence of the *S. aureus*. Indeed, the researchers found in a mouse model of *S*. *aureus* infection that *B*. *subtilis* inhibits QS-regulated gene expression through the production of fengycins, a class of lipopetides with a similar structure to AIP derived from *S*. *aureus* [194]. This work further supports a potential link between QS molecules in healthy and diseased states. It should be noted here that many GI diseases establish after infection with pathogenic bacteria (post-infectious IBS) and that disease conditions are also frequently associated with changes in the gut environment including increased gut pH, altered bile acid pools, increased immunoglobulin concentrations, and the presence of opportunistic pathogens [195,196]. This, in turn, is likely to affect the amount and composition of QS signals derived from the microbiota (Section 2), but it remains to be established whether these pathophysiological changes in the GI environment originate from the host or require the microbiota.

QS itself might also change the composition of the microbiota and/or the expression of microbial metabolites. Thus, in an in-vivo situation, it is hard to disentangle this relationship between QS-molecule-induced changes in the microbiome or their potential direct effects on the host. From ex-vivo studies, however, it is becoming evident that cells aside from immune cells and epithelial cells, such as neurons, can respond to QS-related stimuli and this may constitute an important mechanism by which pathogens can modulate the host response and induce symptoms in the GI tract [52,183,185]. Symptoms of neuronal dysfunction (visceral pain, nausea, and GI dysmotility) alongside increased permeability and inflammation, are hallmark symptoms of GI disease including Irritable Bowel Syndrome (IBS) and Inflammatory Bowel Disease (IBD).

## 5. Perspective

Whilst QS may represent a promising target for the treatment of infectious diseases [29], the inhibition of quorum sensing might simultaneously negatively impact indigenous bacteria. Further, it has been shown that whilst targeting QS systems in pathogens may reduce toxin production and acute lethality, it can, at the same time, reduce immune stimulatory effects that may lead to persistent chronic infection. This highlights the need for a better understanding regarding the regulation of QS and the interaction of different QS systems in an intestinal environment. Unfortunately, current-omics approaches do not allow the study of the growth stage of bacteria in the gut with certainty, and whether specific QS-regulated genes are activated or inhibited. QS in resident bacteria is understudied, and there are several questions yet to be answered. For example, is the cell density in the intestine high enough to trigger QS activation? It would appear so, since AHL, AI-2, and AIP concentrations can be measured [29,197] and are hypothesized to contribute to the cross-inhibition between species as well as niche generation. Questions also remain regarding host-derived quorum quenching molecules or mimetics that could potentially modulate bacterial quorum sensing, which, in turn, could either reduce toxin production in pathogens or stimulate bacteriocin production in commensals.
